# Congenital constriction ring syndrome with foot deformity: two case reports

**DOI:** 10.4076/1757-1626-2-6696

**Published:** 2009-06-26

**Authors:** Korhan Ozkan, Koray Unay, Bora Goksan, Kaya Akan, Nadir Aydemir, Namik Kemal Ozkan

**Affiliations:** 1 Department of Orthopedics and Traumatology, Goztepe Research and Training HospitalIstanbulTurkey; 2 Department of Orthopedics and Traumatology, Medical Faculty of Istanbul UniversityIstanbulTurkey

## Abstract

**Introduction:**

Congenital peripheral constriction ring originating from soft tissues of the leg that is characterized with compression in the soft tissue usually involving the deep fascia surrounding the leg at the time of birth is occasionally observed in lower extremity. At the region of the constriction, fractures of tibia and fibula and foot deformities like clubfoot can be observed.

**Case presentation:**

In our report, 6-month and 8-month old infants with congenital constriction band and ipsilateral clubfoot were presented. They were treated with multiple Z plasties for their constricting bands and Ponseti method of serial casting for their clubfoot deformities.

**Conclusion:**

Congenital-constricting bands can be effectively released with multiple Z plasties. Ponseti method of correcting club foots of various causes can be applied to club foot deformities accompanying constricting bands.

## Introduction

This condition is named as either congenital peripheral constriction rings originating from soft tissues of the leg or as streeter band is occasionally observed in lower extremity [1]. It is characterized with compression in the soft tissue usually involving the deep fascia surrounding the leg at the time of birth. Lymphatic vessels and superficial vascular circulation is usually partially obstructed. At the distal side of the constriction, oedema and cyanosis could be seen. At the region of the constriction, fractures of tibia and fibula and foot deformities (clubfoot) can be observed. Unlike congenital pseudoarthrosis, the fractures can be healed by means of releasing the constriction without need of an operation. According to Patterson [2] constriction ring can be classified into four subgroups:

- Simple constricting ring,
- Constricting ring with deformity of distal part,
- Constricting with fusion of distal parts,
- Complete intrauterine amputation.

Results were graded as good, fair, or poor, based on pain, residual deformity, need for special shoes, inserts, or braces, presence or absence of calluses, and limitation of activities. Children that had no functional limitations, no pain, and little or no residual deformity were considered to have a good result. Results were considered fair if the child had occasional pain, mild to moderate residual deformity, or limitations with strenuous activities or sports. Poor results were in patients who required amputation, with severe pain, with severe limitations in nonstrenuous activities, or residual weakness requiring a brace for ambulation [3].

## Case presentation

**Figure 1.**
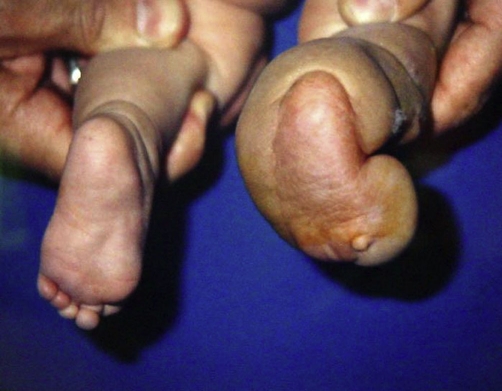
Baby’s foot seen after correction with ponseti method.

Both of our cases have constriction rings on their right legs, mild lymphoedema in the distal region and club foot deformity on the same side (Zone 2 and Grade 3). They were 6 months old Turkish boy and 8 months old Turkish girl respectively. Initially foot deformities of both patients have been corrected by Ponceti casting method (Figure 1). Start was given by correcting cavus component of the deformity, through serial castings forefoot was rendered to supination and the first metatarsi to dorsiflexion. As a result of corrected arcus, forefoot and hindfoot were observed to be in the same alignment. Pronation of the forefoot was carefully avoided. For the correction of varus and adduction deformity, the foot was brought to abduction by asserting a force with our thumb to the talar head in the supination position over the cast.

In the following castings, it was observed that talar head has been reducted and covered by os naviculare and that supination also decreased as the foot was put in abduction position. However, the foot was never brought to pronation.

It was also observed that the foot was plantigrade just as it was brought to complete abduction and that heel varus was corrected with coming of evertion simultaneously. During the manipulation, for the complete correction of the calcaneal alignment, an extra care was given to not to touch to calcaneocuboid joint or the heel. The casting was applied after hand correction, which lasted for 2-3 minutes. In both cases, synthetic cast (soft cast 3M) was used as above the knee. The family was informed about the circulation control and was told to remove the casting in a suspicious situation. The serial casting was performed in 7 days period. For each case seven castings were used. After the correction of foot adduction and heel varus, equinus deformity was corrected by making dorsiflexion of the ankle. Upon the permanent equinus deformity, we performed tenotomy of achilles tendon under general anesthesia.

The casting applied following the tenotomy remained for 3 weeks. After the removal of the last casting, specially designed shoes for the prevention of relapse, we applied Dennis Brown foot braces in the position of abduction at 70° and dorsiflexion at 15-20°. The brace was initially used all day long for a period of 3 months and then after, only during sleep [4].

Six months following the tenotomies we performed the operation of constriction bands. After the resection of soft tissue and deep fascia, both of two constriction bands were released at the same session and corrected by Z plasty procedure with multiple parallel incision of 60° to each other [2].

**Figure 2.**
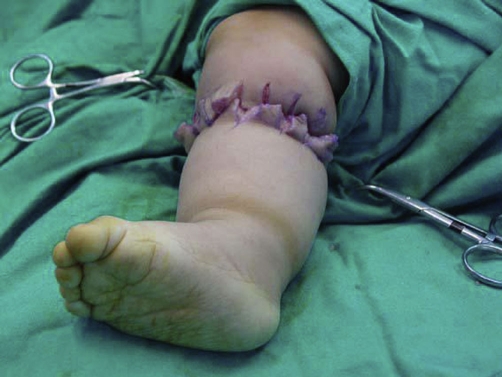
Multiple Z plasties are performed.

**Figure 3.**
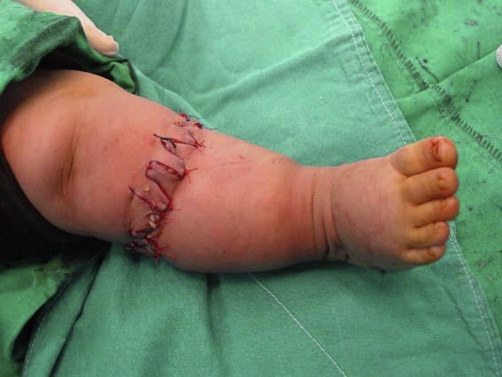
After correction of constricting ring.

**Figure 4.**
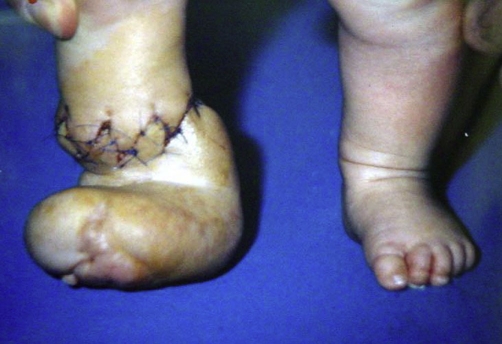
Second baby’s foot corrected with multiple Z plasties.

Tissue necrosis was observed in none of our patients and after 1 month from the operation constriction bands healed without any wound complication (Figures 2,3 and 4). The patients after 1 year of follow up had good results.

## Discussion

The prevalence of clubfeet with constriction bands ranges from 12 to 56 % [5-7]. A series by Cowell and Hensinger reported 14 patients with clubfeet among 25 patients with congenital constriction band syndrome [8]. The location of constriction bands are divided into 4 zones. Zone 1 bands occur between the greater trochanter and the knee. Zone 2 bands occur between the knee and the ankle. Zone 3 bands occur between the ankle and metatarsophalangeal joints. Zone 4 bands are limited to toes. Severity of bands is also considered. Grade 1 bands are subcutanaous, not to the level of fascia. Grade 2 bands are to the level of fascia and not compromise the circulation to the distal extremity. Grade 3 bands are to the level of fascia, such that lymphedema or circulatory compromise necessitates surgical release. Grade 4 bands include all congenital amputations [3]. Both of our cases have constriction rings on their right legs, mild lymphoedema in the distal region and club foot deformity on the same side (Zone 2 and Grade 3). They were 6 and 8 months old respectively. Although clubfeet associated with congenital annular constricting bands are rigid and treated with surgically in the previous reports, the Ponseti method of serial casting to gradually correct the deformity, combined with a percutaneous tenotomy of the Achilles tendon to correct ankle equinus followed by several years of bracing to maintain the correction, has gained widespread popularity in recent years for the treatment of idiopathic clubfoot and is becoming increasingly more widely used [9,10] and our successful results with this conservative method of Ponseti may prevent unnecessary operations in this patient group. Although the clubfeet in the present study were well corrected after 1 year of follow-up, longer follow-up is necessary to assess the continued risk of recurrence and to allow for more accurate recommendations regarding the length of time necessary for brace wear.

## Conclusion

Lower extremity deformities due to congenital constriction bands can be well corrected by reasonable planning and effective operative procedures and congenital constricting bands can be effectively released with multiple Z plasties. Also ponseti method of correcting club foots of various causes can be applied to club foot deformities accompanying constricting bands.

## Abbreviation

None.
